# Characterization of interstitial diffuse fibrosis patterns using texture analysis of myocardial native T_1_ mapping

**DOI:** 10.1371/journal.pone.0233694

**Published:** 2020-06-01

**Authors:** Hossam El-Rewaidy, Ulf Neisius, Shiro Nakamori, Long Ngo, Jennifer Rodriguez, Warren J. Manning, Reza Nezafat

**Affiliations:** 1 Cardiovascular Division, Department of Medicine, Beth Israel Deaconess Medical Center and Harvard Medical School, Boston, Massachusetts, United States of America; 2 Department of Computer Science, Technical University of Munich, Munich, Germany; 3 Department of Radiology, Beth Israel Deaconess Medical Center and Harvard Medical School, Boston, Massachusetts, United States of America; Scuola Superiore Sant'Anna, ITALY

## Abstract

**Background:**

The pattern of myocardial fibrosis differs significantly between different cardiomyopathies. Fibrosis in hypertrophic cardiomyopathy (HCM) is characteristically as patchy and regional but in dilated cardiomyopathy (DCM) as diffuse and global. We sought to investigate if texture analyses on myocardial native T_1_ mapping can differentiate between fibrosis patterns in patients with HCM and DCM.

**Methods:**

We prospectively acquired native myocardial T_1_ mapping images for 321 subjects (55±15 years, 70% male): 65 control, 116 HCM, and 140 DCM patients. To quantify different fibrosis patterns, four sets of texture descriptors were used to extract 152 texture features from native T_1_ maps. Seven features were sequentially selected to identify HCM- and DCM-specific patterns in 70% of data (training dataset). Pattern reproducibility and generalizability were tested on the rest of data (testing dataset) using support vector machines (SVM) and regression models.

**Results:**

Pattern-derived texture features were capable to identify subjects in HCM, DCM, and controls cohorts with 202/237(85.2%) accuracy of all subjects in the training dataset using 10-fold cross-validation on SVM (AUC = 0.93, 0.93, and 0.93 for controls, HCM and DCM, respectively), while pattern-independent global native T_1_ mapping was poorly capable to identify those subjects with 121/237(51.1%) accuracy (AUC = 0.78, 0.51, and 0.74) (P<0.001 for all). The pattern-derived features were reproducible with excellent intra- and inter-observer reliability and generalizable on the testing dataset with 75/84(89.3%) accuracy.

**Conclusion:**

Texture analysis of myocardial native T_1_ mapping can characterize fibrosis patterns in HCM and DCM patients and provides additional information beyond average native T_1_ values.

## Introduction

Myocardial tissue characterization via tissue relaxometry has emerged as a powerful cardiovascular magnetic resonance (cardiac MR) imaging tool to investigate myocardial tissue composition[1]. In the presence of interstitial fibrosis, native myocardial T_1_ time will change and can be measured using T_1_ mapping sequences. T_1_ mapping has been used to distinguish between healthy and diseased myocardium in a wide variety of cardiac diseases[2–5], showing elevated native T_1_ values in patients with hypertrophic cardiomyopathy (HCM)[3,5] and dilated cardiomyopathy (DCM)[6,7] including a strong correlation with extracellular collagen deposition in the latter[6]. Furthermore, recent studies demonstrated the prognostic role of abnormal native T_1_s in HCM and DCM patients[7–10]. Despite differences in global native T_1_ values among cohorts with different cardiomyopathies, there is considerable overlap in global T_1_s[2,4,11] although myocardial fibrosis patterns differ significantly. For example, although myocardial fibrosis in DCM patients is predominantly diffuse[12] and in HCM patients more regional and patchy[13,14], current T_1_ mapping techniques based on the mean T_1_ value[8,10] do not capture these differences. Therefore, there is an unmet clinical need for novel imaging biomarkers to better quantify differences in fibrosis patterns.

Cardiac MR images may contain information that is not being extracted by the current standard image analysis workflow. For example, signal variation in cardiac MR images may contain additional information reflecting underlying pathophysiology[12–14] that is not being quantified. Radiomics[15] and texture image analysis have been recently applied to cardiac MR images[16–20] to extract new quantitative features that may provide diagnostic information. That is, radiomics quantitatively extract high-dimensional feature to differentiate images beyond mean signal value such as signal heterogeneity[17]. This process is usually followed by a selection of independent descriptors that best describe the features. Baessler et. al.[18] demonstrates that texture analysis on non-contrast T_1_-weighted images can detect myocardial tissue alterations in HCM patients with excellent accuracy at differentiating between normal and HCM. Shao et. al.[19] also shows that texture analysis of native T_1_ maps can differentiate between DCM and control subjects. Similarly, Neisius, et. al. demonstrates that texture analysis can differentiate between HCM and hypertensive heart disease patients where a set of six texture features extracted from cardiac T_1_ maps can provide an accuracy of 80% in an independent testing dataset using support vector machines classifier[16]. While these studies demonstrate the potential of texture analysis to diagnose different cardiomyopathies, they do not indicate whether texture analysis can be used as an alternative analysis approach to elucidate differences in tissue compositions.

In this study, we propose to characterize fibrosis patterns via texture analysis on native T_1_ mapping to establish disease-specific features that reflect phenotypic differences of interstitial diffuse fibrosis among HCM, DCM, and control cohorts. We hypothesize that textural analysis of native T_1_ maps can highlight differences in interstitial diffuse fibrosis patterns between HCM and DCM regardless of their functional or morphological parameters.

## Materials and methods

### Study population

We prospectively recruited 321 subjects (55±15 years, 70% male) between July 2014 and March 2018 at Beth Israel Deaconess Medical Center and retrospectively performed radiomic image analyses. The study participants consisted of consecutive patients referred for a clinical cardiac MR exam with suspected or known cardiac disease and healthy volunteers (n = 21) that both meet the criteria described below. The study was approved by the Beth Israel Deaconess Medical Center’s Institutional Review Board (Protocol Number: 2001P-000793). Written consent was obtained. Patients were consented during their CMR scan appointment and research subjects were additionally contacted via advertisement.

The inclusion criteria for the three patient groups were based on established diagnostic criteria and cardiac MR measurements[21–25]. HCM was diagnosed by one of two ways: normal LV cavity size with wall thickness ≥15 mm[21], or a wall thickness above the normal range (≥12 mm for men and ≥11 mm for women[22]) in the presence of high clinical suspicion (i.e. gene carrier and/or HCM family history + LV wall thickness ≥13 mm, etc.), both not explained by loading conditions[21]. DCM was defined as an increase in LV volume (LV end-diastolic volume/body surface area >105 ml/m^2^ for men and >96 ml/m^2^ for women[23]) with coexisting reduction in LV systolic function (LV ejection fraction <53%[25]), and absence of subendocardial-based late gadolinium enhanced (LGE) patterns[24]. Control group subjects (n = 65) had normal cardiac dimensions/volumes, normal cardiac function, and absence of late gadolinium enhancement in common consisted of 21 healthy adult subjects free of cardiovascular disease/intervention and 44 subjects referred for a clinical cardiac MR exam for suspected cardiovascular disease. In the latter group, a review of medical records showed no diagnosis of cardiac disease.

Subjects were excluded from analyses secondary to an established diagnosis of amyloidosis, iron deposition or Anderson-Fabry disease, evidence of inflammatory processes in the myocardium or pericardium, and history of ST-segment elevation myocardial infarction. Part of this dataset (~55%) was previously reported[4,5,16,26,27].

**Fig 1:**
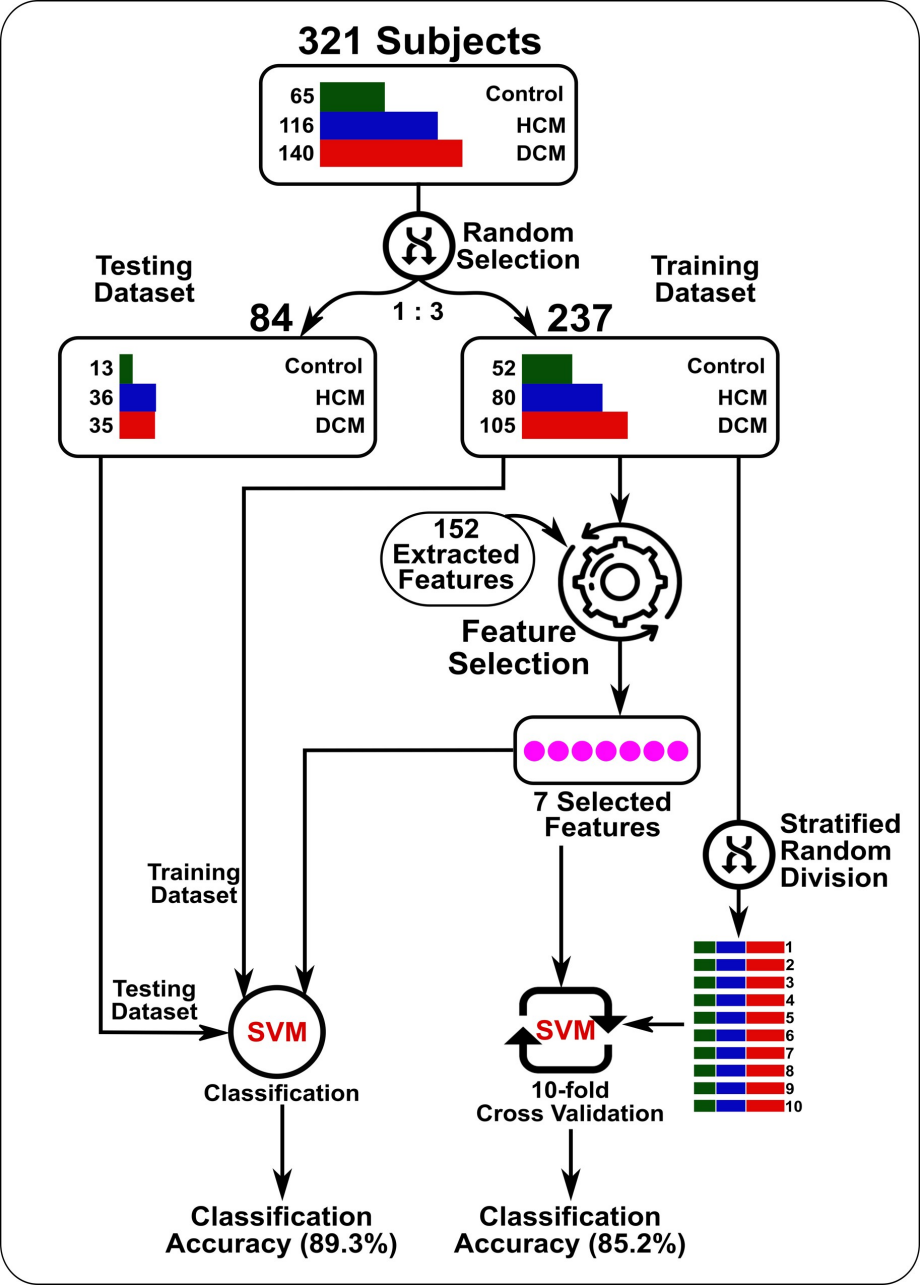
Patient flowchart for the training and testing datasets. The feature selection process was performed only on the training dataset to produce 7 selected features. The selected features were validated on the training datasets using a 10-fold cross-validation strategy on the SVM classifier. In testing, the whole training dataset was used to train an SVM classifier to identify all subjects in the testing dataset.

The dataset was randomly divided into two groups: training and testing subsets (237 and 84 subjects with a ratio of ~3:1, respectively). Feature selection and validation were performed on the training dataset, while the testing dataset was used to assess the generalizability of the final selected features for other subjects (Fig 1).

### Image acquisition and pre-processing

Imaging was performed on a 1.5T Philips Achieva system (Philips Healthcare, Best, The Netherlands) with a 32-channel cardiac coil. In each subject, T_1_ maps were acquired at 5 slice locations covering the LV from the base to apex using a free-breathing slice-interleaved T_1_ (STONE) sequence with the following parameters: TR/TE = 2.7/1.37 ms, FOV = 360×351 mm^2^, acquisition matrix = 172×166, pixel-size = 2.1×2.1 mm^2^, linear ordering, SENSE factor = 1.5, slice thickness = 8 mm, slice gap = 4 mm, bandwidth = 1845 Hz/pixel, diastolic imaging, and flip angle = 70°. The T_1_ map of each scan was estimated by pixel-wise curve fitting using a 2-parameter fit model. Motion correction was performed using the Adaptive Registration of Varying Contrast-Weighted Images for Improved Tissue Characterization (ARCTIC) method[28].

Endocardial and epicardial contours were drawn manually on T_1_ maps of all patients by a single observer (HE with 5 years of experience). To assess intra- and inter-observer variability, the contours were re-drawn by the same observer and an additional observer (UN with 10 years of experience) on a subset of images (84 subjects of the testing dataset) within 6 months from the original drawings. Both observers were blinded to the clinical information and patient data.

**Fig 2:**
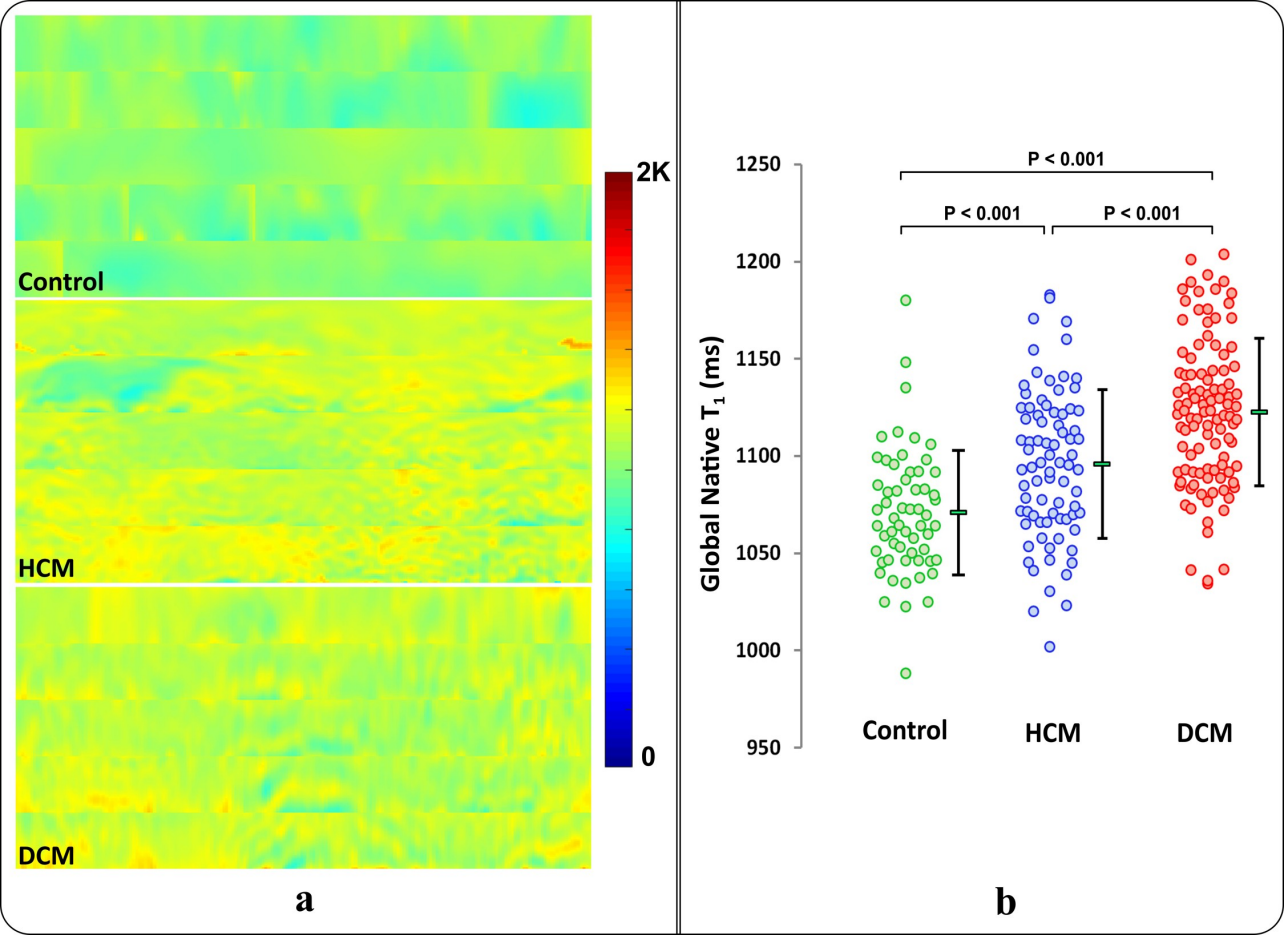
Myocardial native T_1_ maps. (a) 5 slices, from base to apex, stacked in a rectangular shape to represent one control, one HCM, and one DCM patient, respectively. Control T_1_ maps are characterized by a smooth homogeneous profile, while HCM T_1_ maps can be identified by patchy patterns in areas with increased wall thickness, reflecting histological changes of the myocardium. DCM T_1_ maps are recognized by irregular scattered patches of increased T_1_ values. (b) Myocardial global native T_1_ values measured over 5 slices for the three cohorts: control (green), HCM (blue), and DCM (red) from the training dataset. Each dot represents data from an individual subject with the corresponding mean and standard deviation for each cohort.

For texture feature extraction, the delineated myocardial T_1_ maps at each slice were transformed to polar coordinates with a standardized rectangular shape of 32×192 pixels. To maintain the same orientation and starting point of all rectangular maps, a landmark point was manually inserted by the user at the inferior insertion point between the left and right ventricles. The myocardial pixels were resampled into a rectangular form in the clock-wise direction using linear interpolation; such that the bottom left corner of each rectangular map matches the inserted landmark point location on the myocardium. Five rectangular T_1_ maps at different slice levels were stacked per patient to provide a single map representative of the whole heart (Fig 2A).

A three dimensional (3D) phase-sensitive inversion-recovery (PSIR) sequence with spectral fat saturation pre-pulses during the end-diastolic phase approximately 15 minutes after administration of 0.1 mmol/kg body weight gadobenate dimeglumine (Multihance, Bracco Diagnostics Inc., Monroe Township, New Jersey, US) was used to obtain LV LGE images. For the control group, visual inspection was used to exclude the presence of LGE. For the HCM group, LGE was quantified using an automated LV contour and LGE area quantification algorithm specifically developed for LGE quantification in HCM patients[29]. For the DCM group, LGE was quantified using a five standard deviation approach and CVi42 (Circle Cardiovascular Imaging Inc. Calgary, Canada). For all groups, the assessment was performed by experienced (level 3 trained) reader and blinded to clinical and laboratory data. Accurate measurements were assured by visual review of all contours and corrected when necessary.

### Texture features extraction and selection

Four sets of texture descriptors were utilized to extract texture features from the rectangular myocardial T_1_ maps. These descriptors capture spatially-dependent and independent pixel statistics, as well as locally-repeated patterns. Features include: histogram-based features, gray-level run-length matrix (GLRLM)[30,31], gray-level co-occurrence matrix (GLCM)[32], and local binary patterns (LBP)[33] sets of feature descriptors (Table 1). A total number of 152 features were extracted. To reduce redundant information and irrelevant patterns, a feature selection strategy based on the sequential forward selection of the extracted features[34] was employed. In this strategy, features that maximize the characterization of disease-patterns among the different cohorts are iteratively included; where 10-fold cross-validation was utilized to calculate the classification accuracy at each iteration. In this step, 7 features were selected to be most representative of disease-specific patterns in the three cohorts.

**Table 1:** Summary of the extracted and selected texture features.

*Texture Feature Group*	*All features*	*Total # features*	*Selected features*
Histogram-based features	Mean, variance, skewness, kurtosis, 5^th^ to 10^th^ high-order central moments	10	Variance
Grey-level run-length matrix (GLRLM)	Short Run Emphasis (SRE), Long Run Emphasis (LRE), Grey-Level Non-uniformity (GLN), Run-Length Non-uniformity (RLN), Run Percentage (RP), Low Gray-Level Run Emphasis (LGRE), High Gray-Level Run Emphasis (HGRE), Short Run Low Gray-Level Emphasis (SRLGE), Short Run High Gray-Level Emphasis (SRHGE), Long Run Low Gray-Level Emphasis (LRLGE), and Long Run High Gray-Level Emphasis (LRHGE) in 4 Directions	44	Grey-Level Non-uniformity, Short Run High Gray-Level Emphasis
Grey-level co-occurrence matrix (GLCM)	Angular Second Moment, Contrast, Homogeneity 2, Entropy, Correlation, Sum of Squares; for 10 displacements in 4 direction	60	-
Local Binary Patterns (LBP)	LBP Histogram features from 1 to 38.	38	LBP(8), LBP(36), LBP(26), LBP(21)

### Data analysis

Selected texture features were combined in one index, the Texture index (T_x_), using the linear regression equation: Tx = β_0_ + β_1_x_1_ + … β_n_x_n_; where x_1_,…x_n_ represents the selected features, and β_0_,…β_n_ are regression coefficients calculated from the dataset. The texture index was used to test the capacity of the quantified patterns to identify subjects in *binary* comparisons (i.e. one-vs-one). The t-distributed stochastic neighbor embedding (t-SNE) method was employed to visualize the ability of the quantified patterns to cluster each cohort on a 2D plane[35].

Four classifiers: linear support vector machine (SVM), radial basis function kernel SVM, k-nearest neighbor (KNN), and ensemble decision trees[36], were utilized to perform *multiclass* classifications[37] for the quantified patterns among the three cohorts using stratified 10-fold cross-validation[38]. All feature vectors were normalized by the mean and variance before training the classifiers. Receiver operating characteristic (ROC) curves were calculated to assess the classification performance. Areas under the ROC curves were compared using the DeLong method[39]. Normality of data distribution was determined using the Kolmogorov-Smirnov test and visual inspection of the Q-Q plots. The two-sided Student’s t-test or the Mann-Whitney U-test was conducted as appropriate for comparison of continuous variables between groups. Analysis of variance or Kruskal-Wallis tests were used as appropriate for comparison of several groups. For comparison of categorical data, the Chi-squared test was employed. Significance was declared at two-sided P-values <0.05. For pairwise comparisons following a three-group inferential test that was significant, a Bonferroni correction was used. Intra- and inter-observer reproducibility of the selected features was tested using intraclass correlation coefficients (ICC) with a two-way mixed-effect model and Bland-Altman analyses. To test the generalizability of the quantified patterns at identifying subjects from T_1_ maps, the same analyses were conducted on the testing dataset.

All described methods and statistical analyses in this work including motion correction, image reshaping, texture feature extraction[40], and classifiers, were implemented on Matlab (version 2014b, The MathWorks Inc., Natick, Massachusetts, United States). Patient characteristics and standard cardiac MR parameters (listed in Table 2) were analyzed using SPSS (version 18.0; International Business Machines Corp., Armonk, New York, USA).

**Table 2:** Cohort characteristics and standard cardiac MR measures of function and anatomy.

	*Control (65)*	*HCM (116)*	*DCM (140)*	*P-Value*
Age, years	53±15	55±14	55±15	0.533
Gender, m (%)	36 (55)	87 (75)	104 (74)	0.105
Systolic Blood Pressure, mmHg	124±15	129±16	116±18^†^^§^	<0.001
Diastolic Blood pressure, mmHg	75±10	77±14	71±13^ǁ^	0.008
Heart Rate, beats/min	67±11	67±10	75±16^‡^^§^	<0.001
Height, m	1.7±0.13	1.72±0.11	1.72±0.15	
**New York Heart Association Function Status**				
II	0	12	14	-
III	0	3	7	-
Caucasian, n(%)	54(83)	70(78)	99(71)	-
Hypertension, n(%)	25(38)	61(53)	50(36)	-
Dyslipidemia, n(%)	34(52)	69(59)	30(21)	-
Diabetes Mellitus, n(%)	3(5)	17(15)	24(17)	-
LVMI, g/ m^2^	44 [36; 54]	71 [57; 90] ^‡^	68 [55; 84] ^‡^	<0.001
LVM/LVEDV, g/ml	0.60 [0.52; 0.71]	0.96 [0.80; 1.23]^‡^	0.52 [0.44; 0.71] ^‡^^§^	<0.001
Maximal Wall Thickness, mm	9 [8; 11]	19 [16; 22]^‡^	10 [9; 13] ^†^^§^	<0.001
LVEDV, ml	134 [110; 167]	146 [125; 168]	267 [220; 317] ^‡^^§^	<0.001
LVEF, %	62±5	65±7^‡^	32±11^‡^^§^	<0.001
LGE, n(%)	0(0)	86(74)	62(44)	-
LGE/LV mass ratio *, %	0.0±0.0	2.0±4.2^†^	5.8±5.5^‡^^§^	<0.001
Gobal Native T_1_ time, ms	1071±32	1096±38^‡^	1123±38^‡^^§^	<0.001

LVEDV, left ventricular end-diastolic volume; LVEF, left ventricular ejection fraction; LVM, left ventricular mass; LVMI, left ventricular mass index.

*When gadolinium quantification was available (n = 78).

‡ P<0.001 when compared with control subgroup

† P<0.01 when compared with control subgroup

§ P<0.001 when compared with HCM subgroup

ǁ P<0.01 when compared with HCM subgroup

## Results

**Fig 3:**
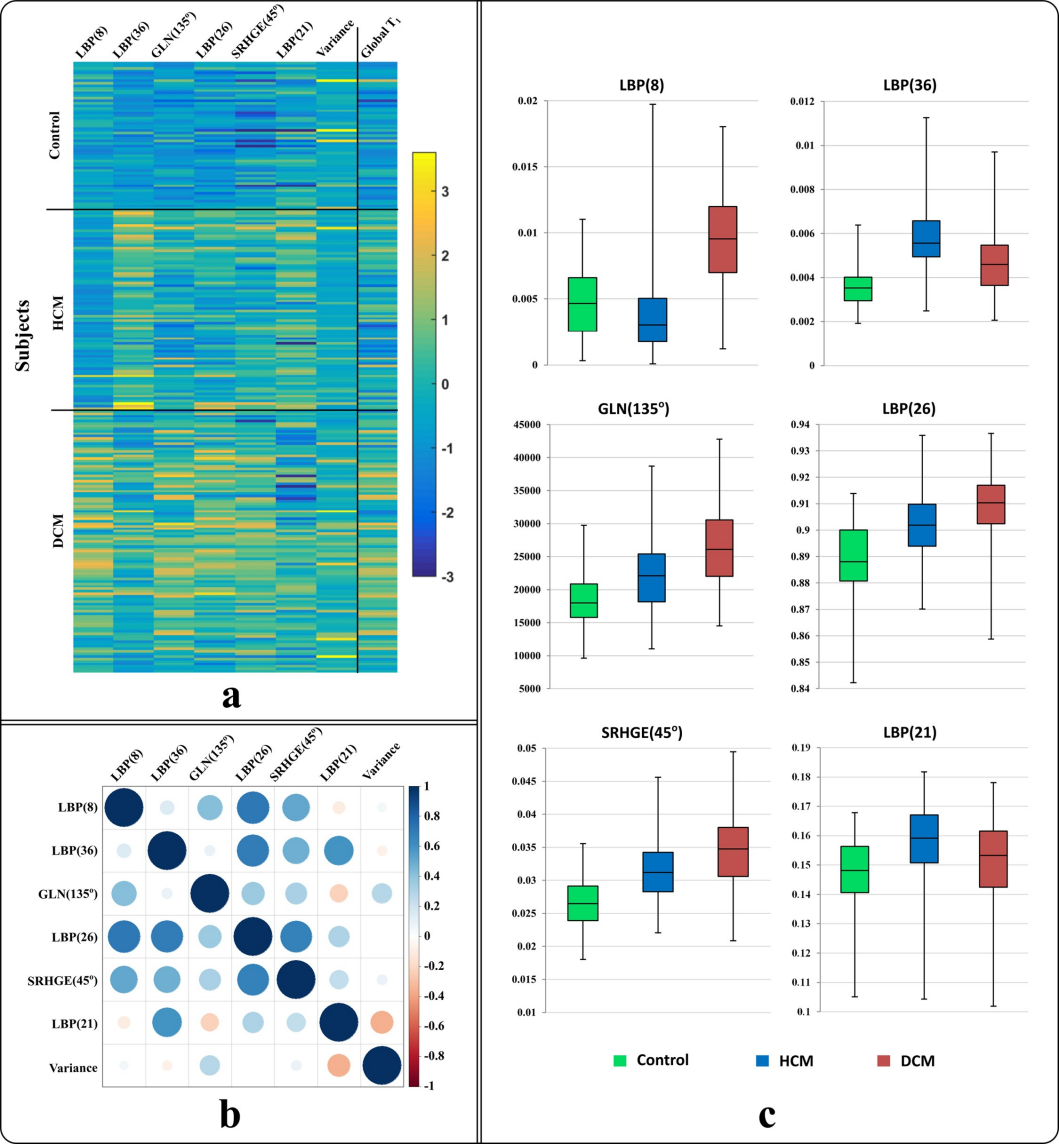
Texture features analysis. (a) Visual comparison of the 7 selected texture features and global native T_1_. Each row represents the feature value for an individual patient. (b) Correlation analysis of the selected texture features. Smaller/lighter-shaded circles indicate lower correlation compared to larger/darker circles. Most of the features have low correlation (i.e. hold independent information). (c) Box-and-whisker plots for the 6 most effective texture features that differentiate disease-specific patterns among 3 cohorts.

Global native T_1_ values varied significantly among the control, HCM, and DCM cohorts (1071±32 vs. 1096±38 vs. 1123±38 ms, respectively; *P*<0.001); however, there was significant overlap among T_1_ values of subjects from different cohorts (Fig 2B). The number of extracted pattern-derived texture features was optimized to reduce overlap among cohorts since most features were highly correlated. Feature selection reduced the original 152 extracted features to only 7 features: 4 LBP histogram features (at indices 8, 21, 26 and 36), 2 GLRLM features (RLN(135°), SRHGE(0°)), and variance of the pixels’ histogram. Selected features were found to capture significantly different patterns among cohorts (S1 Table). We visually compared the selected texture features and global native T_1_ values of the myocardium in all patients, and graphically represented the correlation strength between selected features (Fig 3A and 3B). Box-and-whisker plots show the behavior of 6 selected features to capture specific patterns from different cohorts; each feature either compresses or shifts the data range in one or more cohorts for better identification of fibrosis patterns in each cohort (Fig 3C).

**Fig 4:**
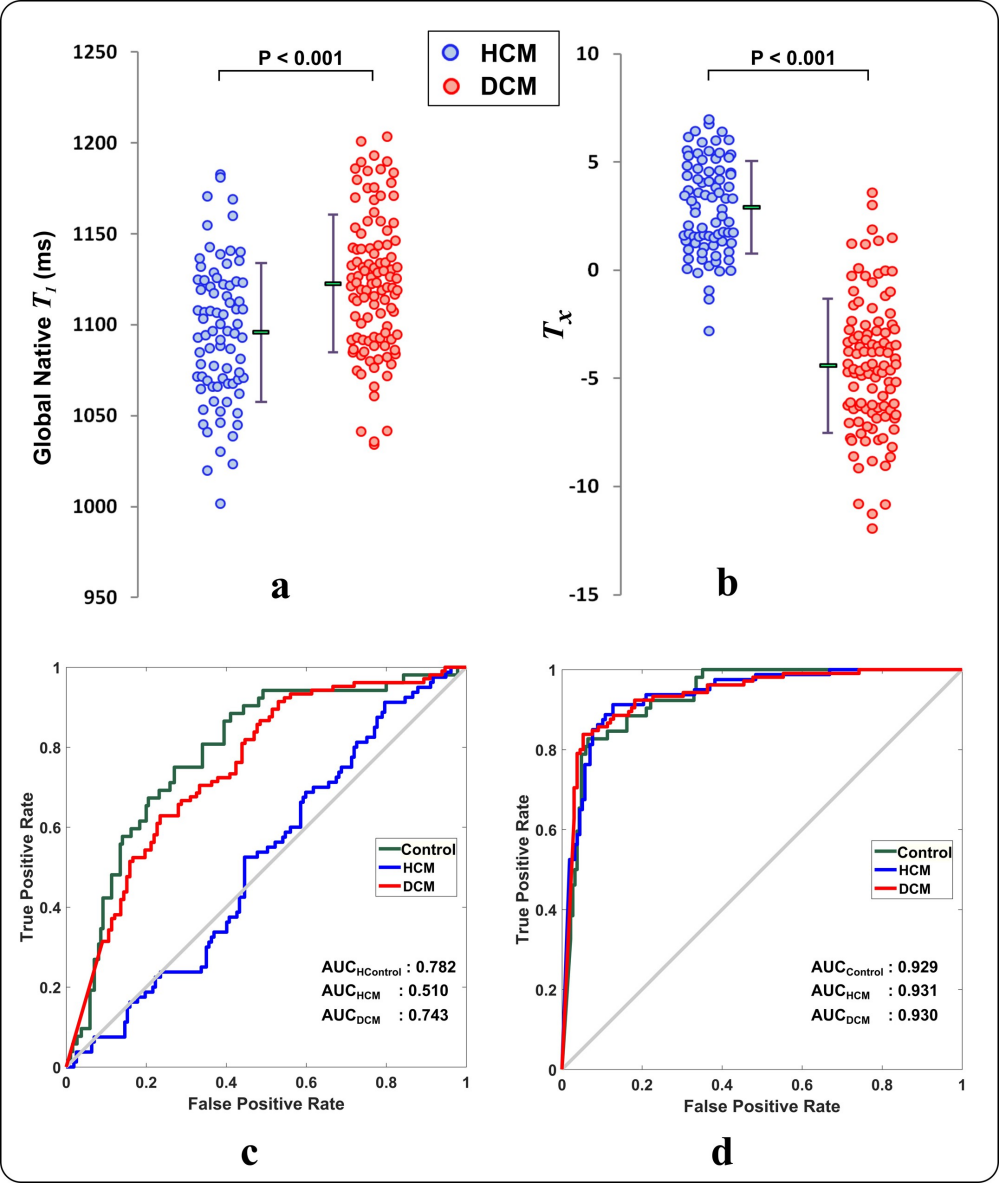
Performance of texture features at identifying fibrosis patterns. (a) Global myocardial native T_1_ values in patients from DCM (blue) and HCM (red) cohorts in the training dataset. (b) The texture index (T_x_) calculated by a linear combination of the 7 selected texture features using a regression model on the training dataset. Each dot represents data from an individual subject. The corresponding mean and standard deviation for each cohort are shown as a line next to its cohort. The texture feature index shows improved differentiation of fibrosis between HCM and DCM when compared to global native T_1_. ROC curves of multi-cohort classification outcomes for (c) global native T_1_ and (d) selected texture features using 10-fold cross-validation on linear SVM in the training dataset.

Combining selected features into one index (i.e. Texture index, T_x_) significantly improved differentiating fibrosis patterns in HCM and DCM subjects of the training and testing datasets (Table 3), relative to pattern-independent global native T_1_ mapping values (*P*<0.001 for all comparisons) (Fig 4A and 4B).

**Table 3:** Texture index (T_x_) values calculated using a linear regression model for binary comparisons (one-vs-one) among 3 cohorts (control, HCM, and DCM) in training and testing datasets.

*Features*	*Control vs. HCM*	*Control vs. DCM*	*HCM vs. DCM*
**Training Dataset**	2.99±2.87 vs. -5.55±4.03‡	3.31±2.7 vs. -4.95±4.08‡	2.9±2.15 vs. -4.42±3.1‡
**Testing Dataset**	2.38±2.51 vs. -6.23±4.27‡	3.48±1.88 vs. -3.21±1.79‡	3.26±1.78 vs. -2.89±2.05‡

‡ P<0.001 when compared with global native T_1_ values.

The performance of the texture analysis to identify fibrosis patterns in a multi-cohort comparison (i.e. one-vs-all) showed the following accuracy: 202/237 (85.23%), 196/237 (82.70%), 193/237 (81.43%), and 192/237 (81.01%) for linear SVM, radial basis function SVM, KNN, and the ensemble tree using 10-fold cross-validation on the training dataset. Based on our preliminary study of different classifiers, linear SVM was used to perform the rest of the comparisons. ROC curves of the texture features showed significant improvement at differentiating fibrosis patterns between cohorts in comparison to pattern-independent global and segmental native T_1_ values (P< 0.001 for all) on linear SVM (Fig 4 and Table 4). When only global native T_1_ was used, the classifier failed to correctly classify the fibrosis pattern in HCM cases, instead of interpreting them as either control or DCM due to extensive overlap in global T_1_ values between control-HCM and HCM-DCM cohorts. Both sensitivity and specificity of the pattern-derived features were higher than global and segmental T_1_ at correctly identifying subjects in all cohorts (Table 4).

**Table 4:** Sensitivity and specificity of global native T_1_ value and pattern-derived texture features to identify subjects in the three cohorts (i.e. control, HCM, and DCM) using multiclass linear SVM.

*Features*		*Control*	*HCM*	*DCM*
**Global Native T**_**1**_	*Accuracy*	*(one-vs-all)* 121/237 (51.1%)		
	*Sensitivity*	0.38	0.31	0.72
	*Specificity*	0.90	0.70	0.62
	*AUC (95% CI)*	0.78 (0.71–0.87)	0.51 (0.44–0.59)	0.74 (0.68–0.81)
**Segmental Native T**_**1**_	*Accuracy*	*(one-vs-all)* 124/237 (52.3%)		
	*Sensitivity*	0.59	0.4	0.58
	*Specificity*	0.81	0.73	0.73
	*AUC (95% CI)*	0.8 (0.74–0.88)	0.62 (0.55–0.70)	0.74 (0.68–0.81)
**Pattern-derived Texture Features**				
10-fold cross-validation on Training Dataset	*Accuracy*	*(one-vs-all)* 202/237 (85.2%)		
	*Sensitivity*	0.79	0.90	0.85
	*Specificity*	0.95	0.90	0.92
	*AUC (95% CI)*	0.93 (0.89–0.98)	0.93 (0.90–0.97)	0.93 (0.90–0.97)
Testing Dataset	*Accuracy*	*(one-vs-all)* 75/84 (89.3%)		
	*Sensitivity*	0.69	0.89	0.97
	*Specificity*	0.97	0.90	0.96
	*AUC (95% CI)*	0.96 (0.92–1.00)	0.93 (0.89–1.00)	0.97 (0.97–1.00)

**Fig 5:**
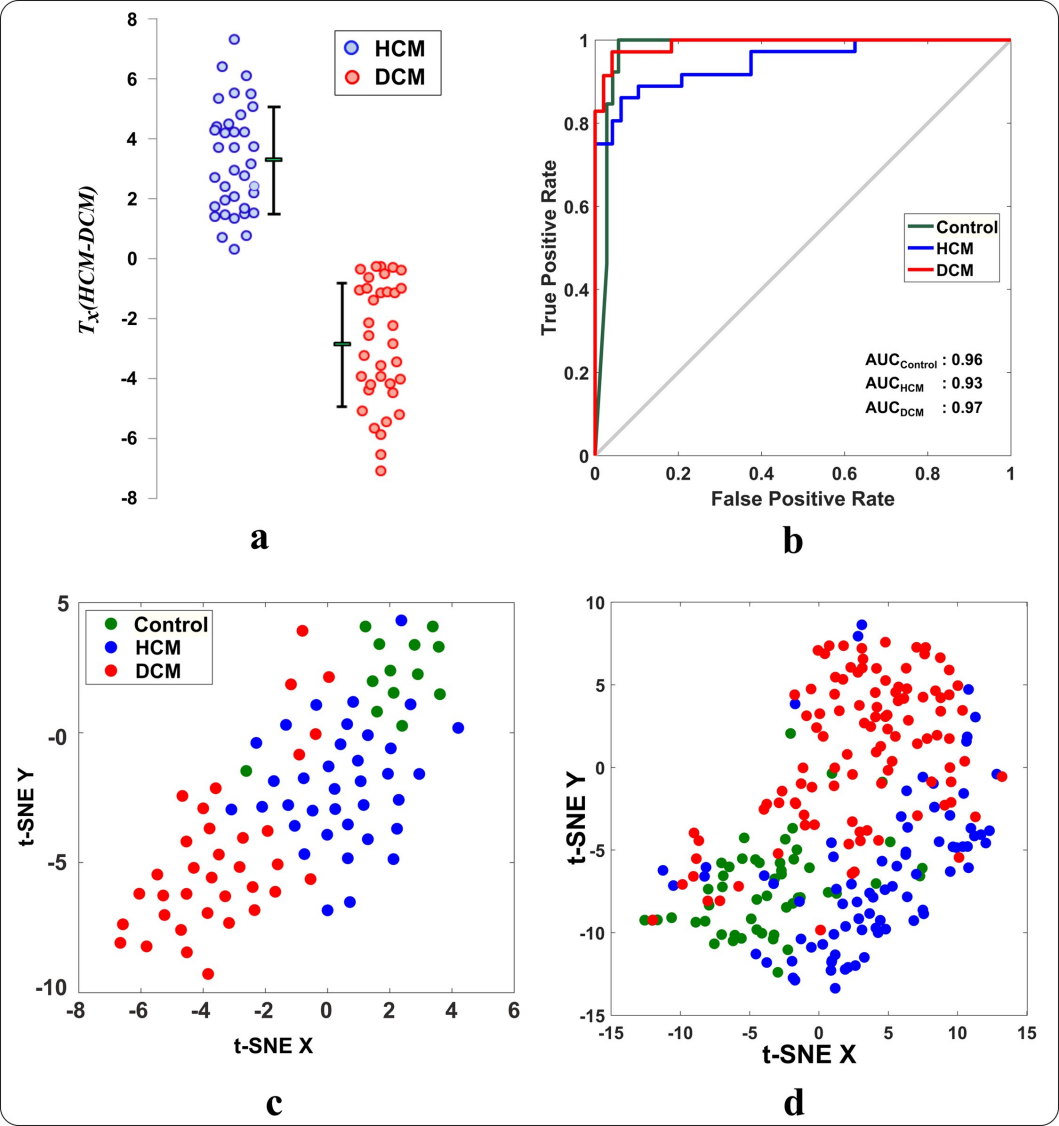
The capacity of the pattern-derived texture features to identify subjects from different cohorts in the testing dataset. (a) the texture index (T_x_) for patients in HCM and DCM cohorts (i.e. calculated by combining the select features using a linear regression model). Each dot represents the T_x_ value for one patient. (b) ROC curves for multi-cohort classification performance of selected features to identify subjects in control, HCM and DCM cohorts in the testing dataset by SVM. The t-SNE visualization of the selected features for all cohorts in (c) the testing and (d) training dataset. Each dot represents pattern-derived features of one subject.

There were no significant differences between training and testing datasets regarding patient characteristics and relevant measurements with the exception of maximal wall thickness (HCM, 19 [17; 23] vs. 16 [14; 21] mm, P = 0.001; DCM, 11 [9; 14] vs. 9 [8; 11] mm, P<0.001). In the testing dataset, the pattern-derived features were generalizable to accurately identify new subjects based on their T_1_ mapping patterns with high sensitivity and specificity values (Fig 5 and Table 4). 2D t-SNE visualization showed the ability of the derived features to separate patients from different cohorts into different clusters with minimal overlap (Fig 5C and 5D). All selected features showed excellent intra- and inter-observer reproducibility (Table 5), and narrow limits of agreement (S1 Fig) except with the exception of T_1_ variance (ICC = 0.7)[41]. T_1_ variance, however, had the smallest contribution in identifying disease-specific patterns among the selected features.

**Table 5:** Intra-class correlation coefficients (ICC) for the intra- and inter-observer reproducibility of the pattern-derived texture features.

	*LBP(8)*	*LBP(36)*	*GLN(135°)*	*LBP(26)*	*SRHGE(45°)*	*LBP(21)*	*Variance*
**Intra-observer**	0.92	0.93	0.96	0.93	0.78	0.77	0.70
**Inter-observer**	0.95	0.98	0.96	0.94	0.95	0.97	0.70

To investigate the effect of T_1_ map spatial resolution on the texture features, the same texture features were extracted from 2 simulated spatial resolutions; where the myocardium in each T_1_ map was resampled to a rectangular form with resolutions of (R_16_ = 16×96 and R_64_ = 64×384 pixels per slice) compared to the current resolution (R_32_ = 32×192). The selected texture features from the three resolution maps were able to identify subjects in HCM, DCM, and controls cohorts with accuracy of 83.6%, 85.2% and 85.7% for R_16_, R_32_, and R_64_ resolutions, respectively, with 10-fold cross-validation on the training dataset and 86.3%, 89.3% and 87.8% in the testing dataset for R_16_, R_32_, and R_64_, respectively. Reducing the resolution slightly decreased the differential capacity of the features, while increasing the resampled spatial resolution to 64×384 achieved similar accuracy as the used resolution of 32×192 pixels.

## Discussion

We demonstrate that texture analysis of myocardial native T_1_ maps can elucidate differences in fibrosis patterns between HCM and DCM patients. We extracted several texture features from native T_1_ maps and subsequently selected independent features that best describe the fibrosis patterns of each cohort in the training dataset. Various classification models were then constructed using the independent information within each feature to improve the differentiation of fibrosis patterns between HCM and DCM.

Standardizing the myocardium in rectangular shape was necessary to allow stacking T_1_ maps from different slices, performing simultaneous feature extraction from multiple slices, and extracting features that are less affected by myocardial geometry and morphology. However, myocardial reshaping could change the shape of the fibrosis pattern and hence affect the capacity of the extracted features to identify different fibrosis patterns. To further investigate this possibility, we conducted additional experiments using an alternative representation that maintains the original myocardial shape for stacking different slices (S1 File). Application of the same feature extraction and selection processes showed a similar capability to identify fibrosis patterns in different cohorts as the reshaped myocardium. The consistent myocardial reshaping maintains the relative differences among different fibrosis patterns and hence did not affect the differential capacity of the features. In addition, correlation analysis showed a low correlation between extracted texture features from the reshaped myocardium and wall thickness (S1 File) indicating that no extracted texture feature from the reshaped myocardium captures geometrical information induced by the reshaping process.

The size of the rectangular myocardium (32x192) was determined based on 6-segments per slice with a segment size of 32x32 pixels. Although this resampling may introduce consistent stretching in the radial direction, the relative differences of texture elements among different cohorts are maintained and should not affect the discriminatory capacity of the extracted features.

Four texture features from the LBP set demonstrated an excellent potential to capture fibrosis-specific patterns. LBP(8) captures DCM-specific patterns on T_1_ maps and its high values significantly distinguish DCM from control and HCM subjects. Two LBP features at histogram indices 36 and 21 capture the distinctive local patchy pattern of HCM and shows significantly increased values in HCM subjects relative to control and DCM subjects. Lastly, the LBP(26) feature had significantly lower values for control T_1_ maps, mainly due to its homogeneous intensity profile.

Furthermore, GLRLM features also captured independent information from native T_1_ maps. GLN(135°) and SRHGE(45°), in particular, added incremental value to identifying different cohorts. GLN(135°) measures the non-uniformity of T_1_ maps and strongly correlated with global native T_1_ values (ρ = 0.98, P < 0.001). Similarly, SRHGE(45°) measured the joint distribution between run length and the pixel value of native T_1_ maps. Despite using GLRLM directional features, the same features calculated at different directions were highly correlated and tended to measure the same pattern.

Similar to LGE scar pattern, texture information of native T_1_ mapping could provide differential diagnosis or prognostic information beyond mean T_1_ values. The current study was not designed to assess the incremental value of texture analysis for the diagnosis of HCM and DCM. A clinical model that includes baseline clinical characteristics, wall thickness, and LGE pattern can already discriminate between DCM and HCM with high accuracy and it is unlikely that the addition of texture information will provide additional diagnostic information. But rather, we demonstrate that differences in diffuse fibrosis distribution and patterns between the two cohorts reflect on T_1_ mapping images and quantifying these fibrosis patterns in form of texture features can differentiate among patients from different cohorts regardless of their functional or geometrical parameters. In addition, differences in interstitial fibrosis pattern may provide additional prognostic information beyond global T_1_ values. For example, patients with more heterogeneous scarring and interstitial fibrosis are more susceptible to ventricular arrhythmia[4,42,43], which may be better quantified via texture analysis of T_1_ maps. Further studies are warranted to investigate the prognostic value of texture analysis of T_1_ maps.

Our study has several limitations. All native T_1_ mappings were acquired at a single center using a STONE sequence on a 1.5T Philips system. Other studies employing different T_1_ mapping sequences, vendors, and field strengths are warranted to assess generalizability. Our population was predominantly male and of Caucasian origin, however, based on consecutive recruitment the cohort was representative for referrals to a tertiary CMR center of a region with a predominantly white population. In HCM histological confirmation of the association between diffuse fibrosis and increased native T_1_ time is required, whilst in DCM the latter is well correlated with the extent of extracellular collagen accumulation[6]. Also, the fixed interslice distance between acquired T_1_-maps leads to altered heart coverage given a disease cohort with altered heart dimensions such as DCM, and its impact on radiomic tissue characterization requires further investigation. Furthermore, our control subjects were partially selected among those referred for a clinical cardiac MR with normal cardiac MR parameters. Further studies should assess the prognostic value of new pattern-derived texture features.

## Conclusion

Texture analysis can extract new reproducible imaging markers from myocardial T_1_ mapping images that have the potential to identify different cardiomyopathies by characterizing disease-specific fibrosis patterns.

## Supporting information

S1 Table Tissue features comparison of healthy, HCM and DCM subjects (median [1st quartile; 3rd quartile]). (DOCX)

S1 Fig (a) Bland-Altman plots for the intra-observer variability of the selected texture features to the manual delineation of LV myocardium. Green lines show the bias, while red lines indicate the limits of agreement (±1.96 Standard deviation). (b) Bland-Altman plots for the inter-observer variability of the selected texture features to the manual delineation of LV myocardium. Green lines show the bias, while red lines indicate the limits of agreement (±1.96 Standard deviation)
(TIF)

S2 Fig Circular myocardium representation. ROI at the myocardium from five slices are stacked from the apex (left) to basal (right) with no reshaping.
(TIF)

S1 File Additional experiments to study the effect of myocardial reshaping on features. (DOCX)

S1 Data (RAR)

S2 Data (XLSX)

S3 Data (XLSX)

S4 Data (XLSX)

S5 Data (XLSX)

## Abbreviations

HCM: Hypertrophic cardiomyopathy
DCM: Dilated cardiomyopathy
Cardiac MR: Cardiovascular magnetic resonance
STONE: Free-breathing multi-slice native myocardial T_1_ mapping using the slice-interleaved T_1_
LV: Left ventricular
GLRLM: Gray-level run-length matrix
GLCM: Gray-level co-occurrence matrix
LBP: Local binary patterns
t-SNE: t-distributed stochastic neighbor embedding
SVM: Support vector machines
KNN: K-nearest neighbor
ICC: Intraclass correlation coefficients
ROC: Receiver operating characteristic
