# Supplementary material for: Characterization of interstitial diffuse fibrosis patterns using texture analysis of myocardial native T1 mapping
Source: PLoS One. 2020 Jun 1;15(6):e0233694. doi: 10.1371/journal.pone.0233694 (PMC7263579; doi:10.1371/journal.pone.0233694)
Supplement: S1 Table — (DOCX) [file pone.0233694.s001.docx]

S1 Table: Tissue features comparison of healthy, HCM and DCM subjects (median [1^st^ quartile; 3^rd^ quartile]).

| **Features** | **Healthy** | **HCM** | **DCM** | **P-value** |
| --- | --- | --- | --- | --- |
| LBP(8) | 21.25 [19.51; 22.07] ×10^-4^ | 12.43[20.16; 16.93]  ×10^-4^ | 25.48[24.47; 57.66]  ×10^-4^‡§ | <0.001 |
| LBP(36) | 5.80[4.89; 10.27]  ×10^-4^ | 6.21[10.07; 24.60]  ×10^-4^‡ | 9.44[8.78; 15.91]  ×10^-4^‡§ | <0.001 |
| GLN(135^o^) | 21.73[28.67; 62.04]  ×10^2^ | 39.49[33.28; 71.00]  ×10^2^‡ | 40.71[44.72; 74.77]  ×10^2^‡§ | <0.001 |
| LBP(26) | 25.43[26.84; 59.07]  ×10^-4^ | 29.12[30.73; 62.01]  ×10^-4^‡ | 41.42[32.81; 97.59]  ×10^-4^‡§ | <0.001 |
| SRHGE(45^o^) | 7.30[11.93; 38.54]  ×10^-3^ | 7.92[7.92; 23.81]  ×10^-3^‡ | 7.98[6.67; 43.66]  ×10^-3^‡§ | <0.001 |
| LBP(21) | 7.58[8.22; 35.48]  ×10^-3^ | 8.32[8.00; 46.50]  ×10^-3^‡ | 10.85[8.25; 40.54]  ×10^-3^‡ | <0.001 |
| Variance | 7.27[15.06; 12.50]  ×10^2^ | 6.36[15.49; 14.60]  ×10^2^‡ | 14.66[16.63; 25.04]  ×10^2^‡§ | <0.001 |

‡ P<0.001 when compared with Healthy Control subgroup

† P<0.01 when compared with Healthy Control subgroup

§ P<0.001 when compared with HCM subgroup

ǁ P<0.01 when compared with HCM subgroup
