# Supplementary figures and images for: Characterization of interstitial diffuse fibrosis patterns using texture analysis of myocardial native T1 mapping
Source: PLoS One. 2020 Jun 1;15(6):e0233694. doi: 10.1371/journal.pone.0233694 (PMC7263579; doi:10.1371/journal.pone.0233694)

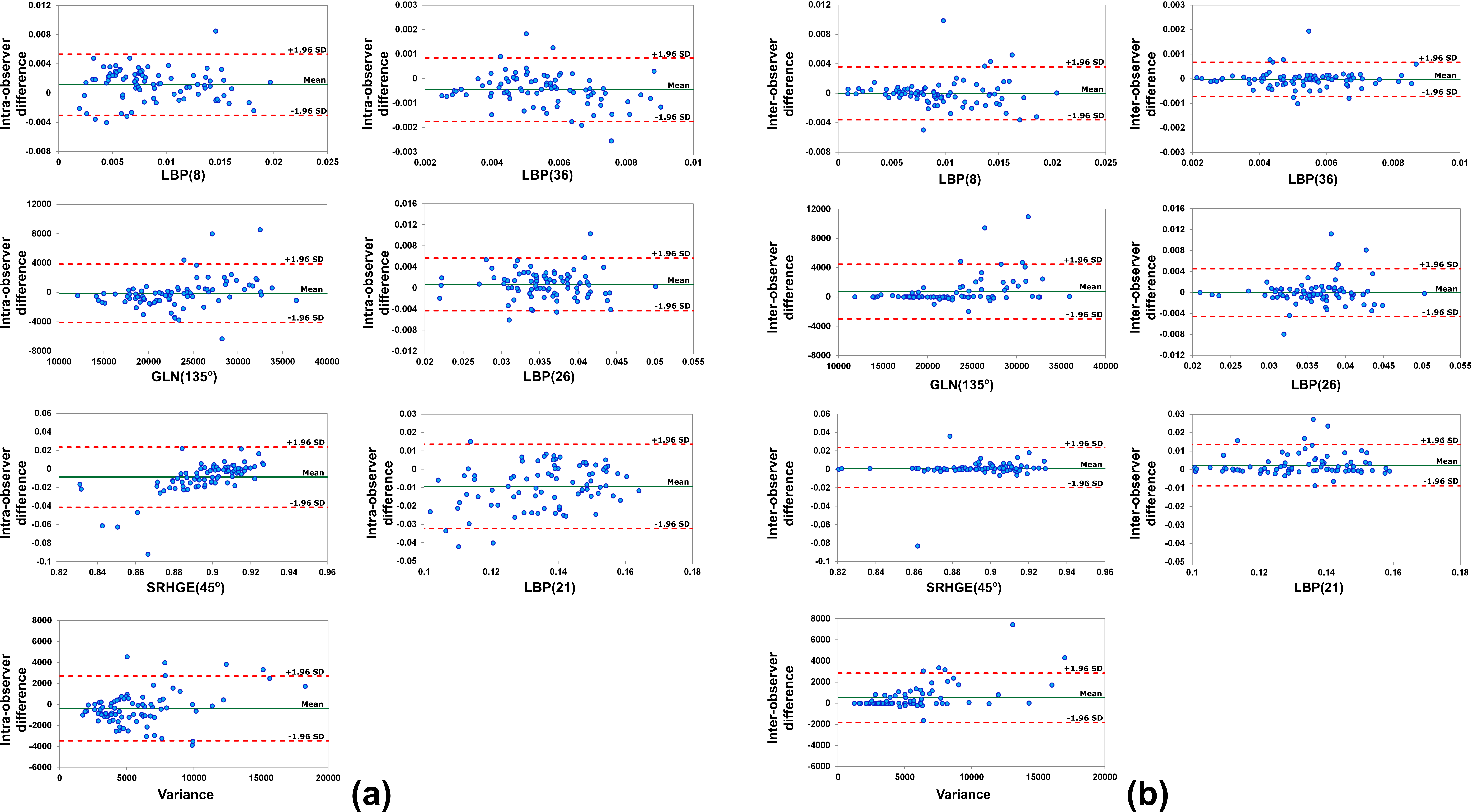

Supplement: S1 Fig — (a) Bland-Altman plots for the intra-observer variability of the selected texture features to the manual delineation of LV myocardium. Green lines show the bias, while red lines indicate the limits of agreement (±1.96 Standard deviation). (b) Bland-Altman plots for the inter-observer variability of the selected texture features to the manual delineation of LV myocardium. Green lines show the bias, while red lines indicate the limits of agreement (±1.96 Standard deviation) (TIF) [file pone.0233694.s002.tif]

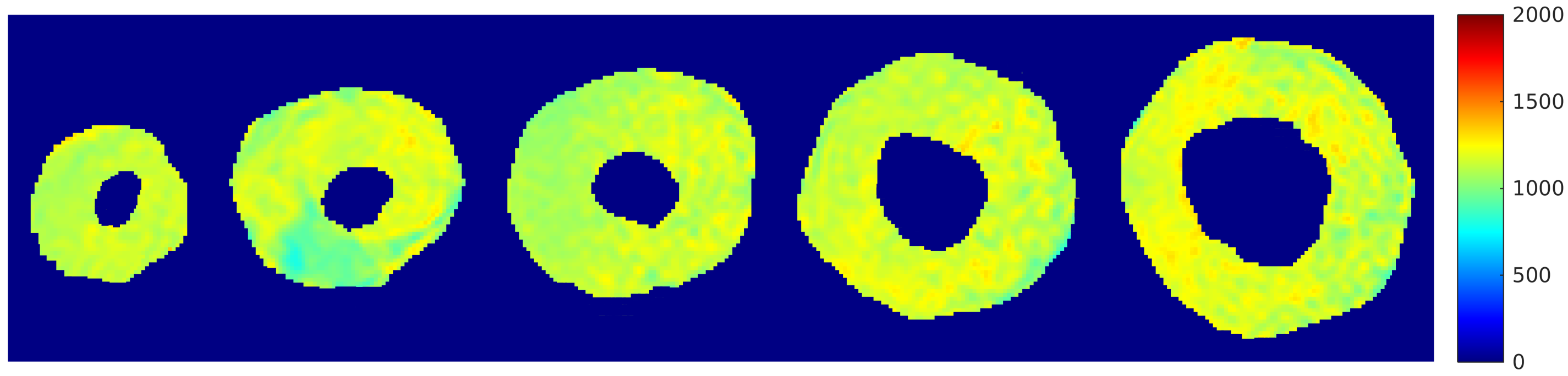

Supplement: S2 Fig — ROI at the myocardium from five slices are stacked from the apex (left) to basal (right) with no reshaping. (TIF) [file pone.0233694.s003.tif]
