# Supplementary material for: Characterization of interstitial diffuse fibrosis patterns using texture analysis of myocardial native T1 mapping
Source: PLoS One. 2020 Jun 1;15(6):e0233694. doi: 10.1371/journal.pone.0233694 (PMC7263579; doi:10.1371/journal.pone.0233694)
Supplement: S1 File — (DOCX) [file pone.0233694.s004.docx]

**Myocardial Reshaping** allows stacking the myocardium from 5 slices and perform simultaneous feature extraction to provide one classification per subject. Although, reshaping the myocardium might change the shape of texture elements (i.e. patterns), the relative differences among these patterns is maintained. To investigate the effect of the myocardial reshaping on the capacity of the texture features to discriminate among different groups, another representation for stacking the myocardium from different slices is also introduced. In this representation, the ROIs from different slices are stacked together in their circular form, as shown below.

Applying the same feature extraction and selection processes on the circular representation of the myocardium showed similar classification accuracy as the features extracted from the reshaped (i.e. rectangular) representation (accuracy: 84.9%; control, c=0.89, 95% CI, 0.84 – 0.96; HCM, c=0.95, 95% CI, 0.92 - 0.99; DCM, c=0.92, 95% CI, 0.89 – 0.97; Sensitivity= 0.8, 0.94, and 0.80; Specificity=0.92, 92, and 95 for control, HCM and DCM, respectively) with different set of selected features; which indicates that the texture information are maintained after reshaping the myocardium and have similar capacity to discriminate among different cohorts.

**Dependency of Texture Features on Myocardial Geometry:** In order to investigate the dependency of the selected texture features on the wall thickness, we conducted a correlation analysis between the 152 extracted texture features and the maximum wall thickness of each subject. The results showed weak correlation between the extracted features and the maximum wall thickness, especially for the selected 7 features: -0.34, -0.01, 0.14, 0.51, 0.14, 0.33, -0.005. Moreover, we used the maximum wall thickness to classify the different cohorts and the accuracy was 63.5% only compared to 85% accuracy achieved by the texture features; which indicates the ability of texture features to collect more information beyond the geometrical information that might be encoded in the extracted features.
